# Ambient Intelligence Context-Based Cross-Layer Design in Wireless Sensor Networks

**DOI:** 10.3390/s141019057

**Published:** 2014-10-14

**Authors:** Yang Liu, Boon-Chong Seet, Adnan Al-Anbuky

**Affiliations:** Department of Electrical & Electronic Engineering, Auckland University of Technology, Auckland 1010, New Zealand; E-Mails: yliu@aut.ac.nz (Y.L.); aalanbuk@aut.ac.nz (A.A.)

**Keywords:** wireless sensor networks, cross-layer optimization, context, ambient intelligence

## Abstract

By exchanging information directly between non-adjacent protocol layers, cross-layer (CL) interaction can significantly improve and optimize network performances such as energy efficiency and delay. This is particularly important for wireless sensor networks (WSNs) where sensor devices are energy-constrained and deployed for real-time monitoring applications. Existing CL schemes mainly exploit information exchange between physical, medium access control (MAC), and routing layers, with only a handful involving application layer. For the first time, we proposed a framework for CL optimization based on *user context* of ambient intelligence (AmI) application and an ontology-based context modeling and reasoning mechanism. We applied the proposed framework to jointly optimize MAC and network (NET) layer protocols for WSNs. Extensive evaluations show that the resulting optimization through context awareness and CL interaction for both MAC and NET layer protocols can yield substantial improvements in terms of throughput, packet delivery, delay, and energy performances.

## 1. Introduction

Wireless sensor networks (WSNs) are an enabling technology of smart environments for ambient intelligence (AmI). In AmI, WSNs perform human-centric sensing where low-level sensor data on users and their surroundings are collated and processed to infer higher-level user context information for context-adaptive AmI applications. The term *context* refers to information that describes the current state or situation of an entity, which can be a person (e.g., user context), place, or object. The high-level user context information is a necessity in AmI applications to deliver personalized services to the users in an intuitive and intelligent way to support their everyday activities. Moreover, we envision such user context information could be harnessed for optimizing the performance of the underlying WSNs through cross-layer (CL) interactions.

By allowing direct information exchange between non-adjacent protocol layers via CL interaction, network performances such as energy efficiency and delay can be optimized [1]. This is particularly important for WSNs where sensor devices are energy-constrained and deployed for real-time monitoring applications. In the current literature, most research on CL optimization for WSNs have focused on interactions between lower layers of the protocol stack, *i.e.*, physical, medium access control (MAC), and network layers [2]. There was also research on CL optimization that considered application requirements, e.g., quality-of-service (QoS) requirements of multimedia applications [3].

Unlike these previous works that either were not concerned with the application layer or used the application to only define the requirements of CL optimization, this paper focuses on how application derived information, *i.e.*, the user context information derived from AmI application, can optimize the underlying WSN performance through CL interactions.

At the system level, an AmI system can adapt its intelligent services to user-related context information. However, its underlying WSN rarely considers AmI context information. If user context information can influence how an intelligent system responds, there is also a possibility for the underlying network to use the user context information. This allows the network protocols to become *smart* by adapting their functionality to user situations.

For the first time, we proposed a generic and customizable CL framework that utilizes AmI context information from application layer for optimizing protocol performance in WSNs [4]. This framework can be applied to any layer of the protocol stack and is sufficiently generic to be customized to different AmI applications. In this paper, we present the framework's architecture in more detail and apply the proposed framework to jointly optimize the backoff behavior of a contention-based MAC protocol, and the path selection of an *ad hoc* On-demand Distance Vector (AODV) based routing protocol for WSNs by adapting their protocol functions in real-time to the user context information inferred from an AmI application.

The rest of the paper is organized as follows. Section 2 outlines related work. Section 3 presents the motivating scenario. Section 4 describes the proposed framework. Section 5 illustrates a use case of the proposed framework by implementing it to optimize two existing protocols at the MAC and NET layers. Section 6 presents and discusses the evaluation results. Finally, Section 7 concludes the paper.

## 2. Related Work

Many existing cross-layer protocols have been designed. However, they often ignore some important information such as context information, which can be relevant for network optimization. This section reviews a number of representative context-aware cross-layer designs in WSNs.

The CIVIC protocol [5] adapts the routing mechanism to the power level of the sensor nodes and context information of a network. CIVIC exploits the *meta* data context information from applications for the purposes of application data security and compression. By adapting its routing to the application level contexts, CIVIC can select paths based on different security and priority levels for efficient data transmissions. However, CIVIC does not specify how it structurally organizes the contexts, and this may limit its applicability.

The energy-efficient Context Adaptive MAC (CA-MAC) protocol [6] for WSNs uses the node buffer states and the priority context of upper layer packets to amend the transmission schedule of a node by putting the node into the sleep mode whenever possible. CA-MAC only considers application level packet priority as a context, and may not behave well under a long burst of high priority packets.

A hybrid TDMA/contention-based context-aware MAC protocol for wireless body area networks is proposed in [7]. The body sensor nodes dynamically adapt its mode of channel access, transmit duration, and sampling rate according to changing human body and environmental contexts. However, this MAC protocol is designed for a simple star topology network, which is not suitable for large-scale WSNs with complex topology and multi-hop communications.

The context-aware cross-layer (CACL) broadcast scheduling scheme for ad hoc networks [8] uses context available locally in a node's MAC layer such as local node density, current state (idle, transmit, receive) and previous transmit duration, to schedule the broadcast of packets from the network routing layer in order to minimize broadcast collisions under heavy load conditions. This scheme does not use contexts that may be available from other layers or from sources that are external to the node.

In the Context-Aware Clustering Hierarchy (CACH) routing protocol [9,10], the WSN is clustered based on the detected environment contexts. A cluster is formed by a group of sensor nodes with their sensor readings in a similar range. However, as sensor readings can be non-uniform throughout the network, the cluster size may differ significantly between clusters, resulting in unbalanced traffic load and energy usage within the network.

In [11], the Context-Aware Multi-path Selection algorithm (CAMS) selects the right number of paths from the available paths for multimedia communication in WSNs. The CAMS only consider the data value from sensors (representing brightness and noise levels) as context information. This can cause problems such as a difficulty in distinguishing between situations with identical data value in different scenarios.

The Reactive Environmental Monitoring Aware Routing (EMA) [12] is an implementation of a generic framework for context aware routing in WSNs. The framework considers the node state, received signal strength, and hop count of a route as context criteria for making routing decisions. However, Reactive EMA only considers node and network level contexts, but not the context information encapsulated in the packets from the application.

In [13], context information is utilized for QoS management in WSNs, where differentiated QoS guarantees can be provisioned to the communication of sensor data according to their spatial and temporal correlations. However, only the location and transmission rate of sensor nodes are considered as context information, which may limit the options available for managing QoS requirements in WSNs.

A cross-layer architecture proposed in [14] supports network level context awareness in communications. Context is categorized into either “local view” context that is inferred from within a sensor node, or “global view” context that is obtained from external of the node. By unifying context representation and cross layer functions, this architecture allows local and global context information to be exchanged to achieve better cross layer optimization outcomes.

It is noted that these previous works were either not concerned with the application layer or used the application to only define the requirements of CL optimization. While context-awareness is a feature found in most of these works, the types of context considered are limited to those at the protocol level, node level, or network level. None of the existing works have considered harnessing the *AmI context*, *i.e.*, user-related context necessary for AmI applications to deliver personalized services to their users in an intuitive and intelligent way, to additionally optimize the performance of the underlying WSNs through cross-layer (CL) interactions, which is the focus of this paper.

## 3. Motivating Scenario

This section describes a common scenario in the AmI domain, and presents the functions of the AmI system and its underlying WSN in the scenario. In addition, it discusses the challenges of optimizing WSN with available context information from AmI system. This scenario depicts an intelligent event notification system for people in an outdoor AmI environment. Users can be notified about the occurrence of physical events in their surroundings that could be relevant to them based on their attributes, such as age and disability status, and the context of the events. This system can be applied to people in all age groups.

**Figure 1.**
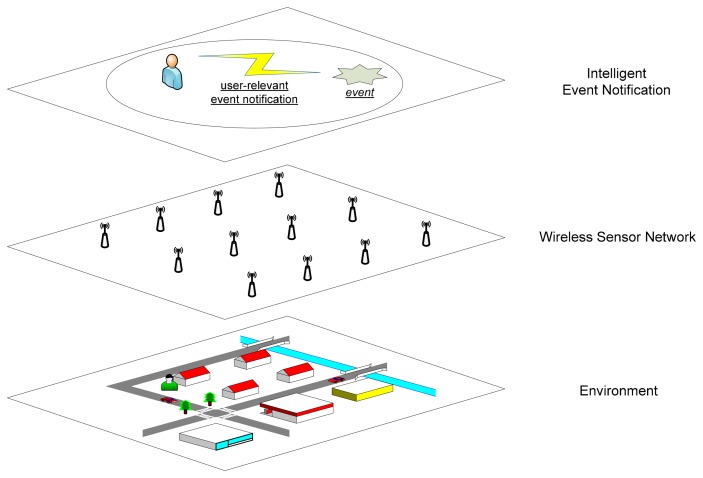
Scenario overview.

Figure 1 shows the scenario of an intelligent event notification system operating over a WSN deployed in an outdoor environment. A WSN is deployed in this environment where some sensor nodes are embedded into inanimate objects, such as buildings and roads, while others could be on mobile objects such as cars and humans (e.g., wearable sensors). The sensor nodes can continuously monitor and detect changes in the physical properties and attributes of their environment. Each person in this environment is assumed to carry a form of smart device, e.g., smart phone, which has an intelligent software agent running on it. The intelligent agent can collect sensor data from the underlying WSN where the person is located, infer events with the collected sensor data, and notify the user about some inferred events that may affect him/her. For instance, a user can receive a notification alert from his smart device when it anticipates an incoming vehicle on the user's movement path.

Consider the case of Sam, a nine year-old boy playing basketball in his backyard. The ball rolls to the street next to the backyard and Sam runs to retrieve it. At the same time, John drives a car that is turning into the street where Sam is located. For this notification system, the sensor nodes in the environment are publishing their data continuously to the VBs, e.g., data from motion sensors along the street, proximity sensors on the backyard fence, and activity sensors worn by Sam. The intelligent agent on Sam's smart device is also receiving the subscribed sensor data from the VBs about the surroundings of Sam based on his location, e.g., the sensor data of the street next to his backyard. With the collected sensor data, the agent can infer the occurrence of possible events that may affect Sam. At this time, the agent infers that a vehicle driven by John may crash into Sam. Hence, the agent declares a state of emergency and the smart device immediately alerts Sam not to cross the street until the vehicle has passed. Simultaneously, while John is driving, his intelligent agent constantly subscribes to receiving sensor data about the streets ahead of his vehicle. In this occurrence, John's smart device notifies him that someone may cross the street ahead, and suggests him to slow down and watch out for the person to avoid any potential incident.

### Challenge of Using AmI Context for WSN Optimizations

The purpose of the WSN in the above AmI scenario is to collect sensor data of the monitored environments and deliver them to the intelligent agents for the inference of possible events related to particular users. A WSN node can only generate raw sensor data, which may not present any meaningful information if the data is not processed further. Therefore, communicating the sensor data from the WSN to the intelligent agents is an important part of the AmI system in order to successfully deduce and notify the user-relevant events on time.

In this scenario, the pub/sub based communication is used, in which data from the same sensor can be subscribed by different intelligent agents at different rates, *i.e.*, frequency of receiving an update of the data, under different situations. For instance, when Sam is detected to be approaching the street, the intelligent agent of his smart device takes the sensor data about vehicles on the street very seriously, and changes its subscription to receive an update of this sensor data more frequently. On the other hand, the same sensor data would be less important to Sam's agent if the ball had not rolled onto the street, and his agent would have just monitor this sensor data at a normal rate.

Therefore, the importance of a piece of sensor data may not be determined by individual sensor nodes, but by an AmI system, such as Sam's intelligent agent in the above scenario. In addition, different agents may perceive the importance of a piece of sensor data differently even at the same time. For instance, Sam's agent will regard the sensor data from the street as highly important, while those of his playmates who remain in the backyard may not. Therefore, there is a need for a mechanism for the sensor nodes to optimize the communication of their data to the AmI systems in different situations.

## 4. Generic Context Aware Cross-Layer Framework

The importance of the sensor data to particular users can be known by the sensor nodes via *reversed* pub/sub communications, where intelligent agents are context publishers that publish their inferred contexts to the VBs, and the sensor nodes are context subscribers that subscribe to particular context based on the sensor nodes' attributes such as location or sensor data type. In other words, a sensor node can subscribe to the context of an event occurring in its area and which requires its sensor data in order to be inferred. In this way, sensor nodes can become AmI context aware, and accordingly optimize the communication of their data to the intelligent agents through cross-layer interaction.

In this paper, a generic CL framework that can be adapted by any context-aware systems is proposed where protocol optimization can be achieved by allowing the inferred AmI contexts to become available to the sensor nodes through a context exchange mechanism, and allowing each node to control its transmission of any outbound data based on the data content and the inferred context of its surrounding. The framework can be implemented in firmware, and each node in the network maintains an instance of the implementation.

This section presents the architecture of the framework, including the functionality and behavior of its constituent components. There are three parts to this framework: (i) communication mechanism for network-wide AmI context exchange; (ii) node architecture for node-level context handling and CL optimization; and (iii) ontology-based context modeling and reasoning mechanism for representing and inferring context within this framework.

### 4.1. Communication Mechanism

The communication mechanism of this framework is based on the data-centric pub/sub paradigm [15]. Under this mechanism, the AmI system is a *publisher* that may publish inferred contexts, while sensor nodes are *subscribers* that may subscribe to published contexts. A VB is a virtual brokerage entity formed by a cooperative group of sensor nodes that share the responsibility of providing context storage and retrieval services. Hence, any AmI context can be disseminated to subscribing sensor nodes for making informed optimization decisions based on situations of their monitored environment.

**Figure 2.**
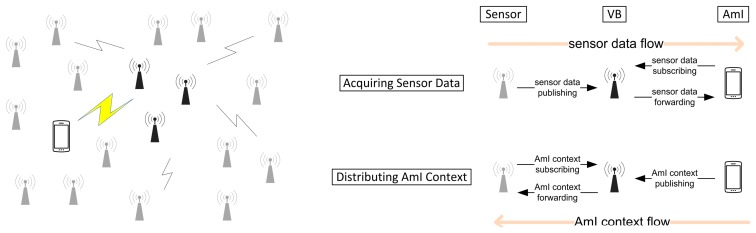
Communication model.

**Figure 3.**
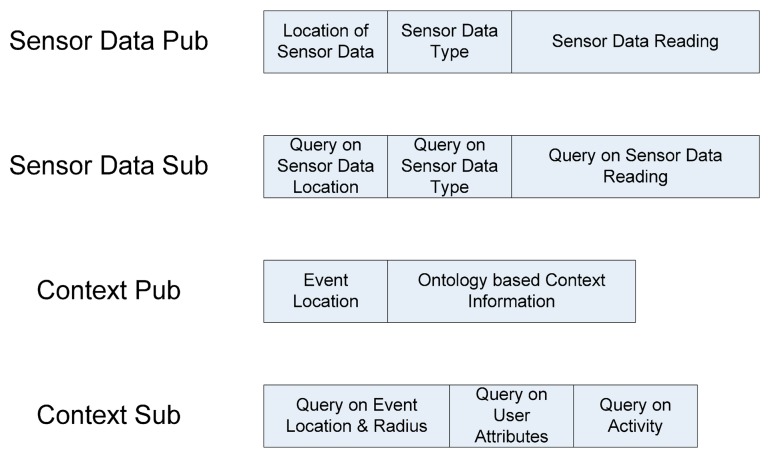
Data structures for bi-directional Pub/Sub.

Figure 2 presents the communication model of this framework. It illustrates the bidirectional information flow: sensor data flow in one direction, and AmI context flow in opposite direction between the sensor nodes and AmI intelligent agents. The data structures for the bi-directional pub/sub communication are shown in Figure 3.

### 4.2. Node Architecture

**Figure 4.**
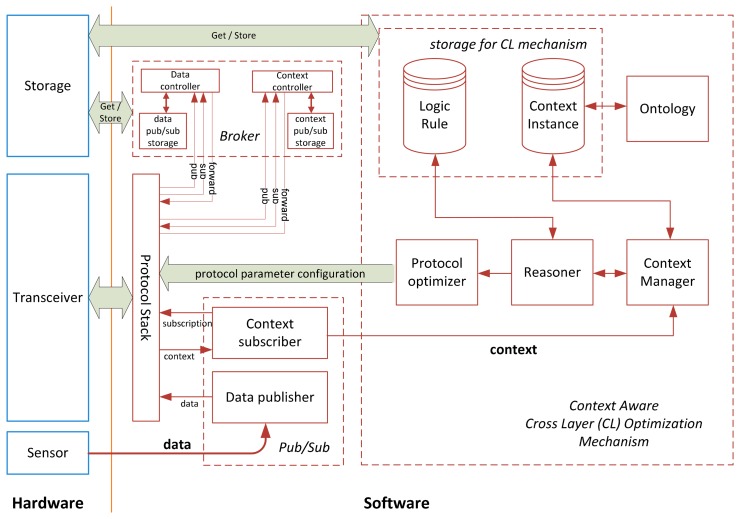
Node architecture.

Figure 4 presents the node architecture of the framework. It illustrates the node-level context handling and protocol adaptation for CL optimization. This architecture has three hardware components, namely sensor element, transceiver, and storage device; and three software modules, namely Pub/Sub control, broker management, and context-aware CL optimization.

The Pub/Sub control module allows a sensor node to perform the roles of both publisher and subscriber, *i.e.*, not only can the sensor node publish its sensed data to the VB (for AmI agents to subscribe and generate higher-level user contexts), it can also subscribe to receive the high-level user contexts from the VB (published by AmI agents and stored on the VB). The broker management module is only used when the sensor node becomes a VB member node. It enables the sensor node to perform brokerage functions such as storing received published data and subscriptions, and forwarding matched published data to subscribers.

The context-aware CL optimization mechanism is the key constituent of this framework. Through context subscription, a sensor node can receive AmI context information, which is stored and later retrieved by the Context Manager for processing. The context information is modeled by an ontology. With some logic rules and a logical reasoning component, a sensor node can interpret the context and configure the protocol parameters for the desired performance.

### 4.3. Ontology-Based AmI Context Model

**Figure 5.**
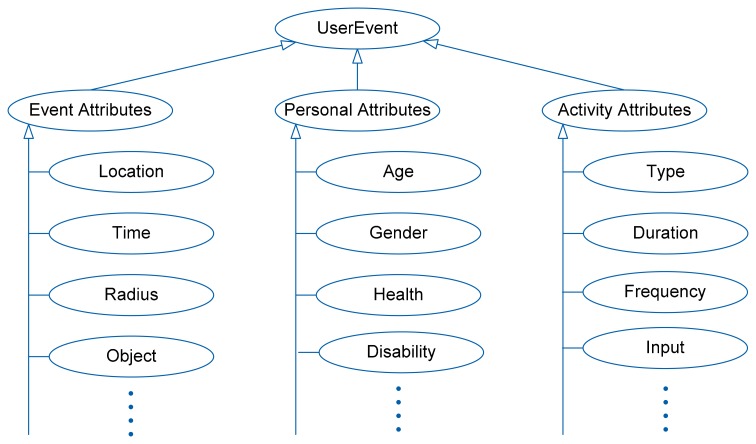
Ontology model for representation of common AmI contexts.

Representing context information requires a modeling method to standardize and formalize information, through which a common understanding of the exchanged (global) AmI context by all sensor nodes can be achieved. In this paper, the representation of common AmI contexts such as user location and activity is shown in Figure 5, which is based on an ontology model derived from our previous work [16].

This ontology model supports context representation for common AmI scenarios. The ontology captures the basic attributes of a user-relevant event, including attributes of the event itself and those of the user (personal and activity attributes) during the event. The class *UserEvent* is the root entity. Each instance of *UserEvent* represents a detected event relating to/affecting a particular user, and is generated by an intelligent agent on behalf of the user. The class *UserEvent* has three descendant classes: *Event*, *Personal,* and *Activity Attributes.* The *Event Attributes* class presents the facts of the event, such as the location and time of event, the event radius (or perimeter), and the status of the objects within the event radius. The *Personal Attributes* class captures the personal facts of the user, such as age, gender, health, and disability status. The *Activity* class encompasses the details associated with the physical activities of the user, such as the type, duration, and frequency of the user's activity (e.g., walk, run, sleep, talk), and the input data (e.g., sensor readings) required for the inference of the activity.

A representation of this Ontology can be described, for example using RDF/XML serialization:
 <owl:Class rdf:ID=“UserEvent”> <owl:Class rdf:ID=“Event Attributes”> <rdfs:subClassOf rdf:resource=“#UserEvent”/> </owl:Class> … <owl:Class rdf:ID=“Personal Attributes”> <rdfs:subClassOf rdf:resource=“#UserEvent”/> </owl:Class> <owl:ObjectProperty rdf:ID=“age”> <rdfs:domain rdf:resource=“#Personal Attributes”> <rdf:range rdf:resource=“xsd:integer”> </owl:ObjectProperty> <owl:ObjectProperty rdf:ID=“disability”> <rdfs:domain rdf:resource=“#Personal Attributes”> <rdf:range rdf:resource=“xsd:disabilitystate”> </owl:ObjectProperty> <owl:Class rdf:ID=“Activity Attributes”> <rdfs:subClassOf rdf:resource=“#UserEvent”/> </owl:Class> …</owl:Class>

The context information of a user-relevant event can be represented by one instance of this ontology. It is important not to make this context model too complex as the sensor nodes are resource-constrained and lack the capacity for complex processing.

#### 4.3.1. Generality of the Model

While the context classes and attributes of the ontology model can be used in different AmI scenarios, each instance of this ontology will be populated by context data that is scenario-specific. Therefore, the ontology model can be scenario-dependent, but only for its context data. Apart from the ontology model, the two other components of the proposed framework: communication mechanism and node architecture described in Sections 4.1 and 4.2, respectively, are scenario-independent, *i.e.*, the same communication mechanism and node architecture can be applied to different AmI scenarios.

To illustrate the generality of the ontology model itself (not its context data), we followed a design evaluation method called *scenarios* [17], which constructs detailed scenarios around the artefact (in this case our ontology model) to demonstrate its utility. Table 1 shows how contexts from four different AmI scenarios in literature: smart home [18], smart office [19], outdoor smart lighting [20], and elderly care [21], can be expressed by the same ontology model.

#### 4.3.2. Scenario-Based Customization

In this section, we illustrate how the proposed ontology-based context model can be customized for the motivating AmI scenario outlined in Section 3. The following assumptions have been made:

- All location information is represented in the form of relative node location defined according to the Anchor-Free Localization (AFL) algorithm [22], *i.e.*, in terms of hop counts to 4 corner reference nodes. A user's smart device is also capable of determining the AFL-based location of the events or users.
- To simplify the context design, personal attributes of a user are represented in terms of a high or low vulnerability state. For instance, both children and elderly are commonly considered as vulnerable individuals in AmI scenarios and therefore are represented with a high vulnerability state in the age-associated attribute. On the other hand, individuals of other ages are represented with a low vulnerability state in this attribute. Disability is another common personal attribute in AmI. People with certain disability types can be more vulnerable than those with other disabilities in a particular AmI scenario. For instance, people with vision or walking impairments are at greater risk in the outdoor scenario presented in Section 3. Therefore, they can be represented with a high vulnerability state in the disability-associated attribute, while those with other disabilities or otherwise healthy can be represented with a low vulnerability state in this attribute.
- Each sensor node has a simple ontology-based context module for performing simple context reasoning tasks. The objective of this reasoning is to determine the importance of the sensor data to the context inference process of the intelligent agents.

A sensor node can use the following first-order logic expression to deduce its data priority context after receiving the event context information generated by the intelligent agents:

*(**eventLocation** (close to the sensor) ∨ **eventRadius** (sensor within the event radius)) ∧ (**personalAge** (high vulnerability state) ∨ **personalDisability** (high vulnerability state)) ∧ **activityInput** (sensor's generated data)* ├ ***SensorData** (Priority, High)*

This expression is used by each individual node to determine the priority context of its sensor data according to the attributes of the event (either the sensor node is located close to the occurrence of the event or the node is within the event radius), personal attributes of the user (age- and disability-associated vulnerability), and activity attributes of the user (input data required for inferring the activity).

For the scenario presented in Section 3, a piece of sensor data is assigned to high priority when the following three characteristics are met:

- The sensor generating the data is close to or within the radius of an event;
- The age or disability attribute of the user related to/affected by the event is in high vulnerability state;
- The sensor data is required for inferring the activity of the user related to/affected by the event.

## 5. Context Aware CL Optimization on MAC and NET Layers

AmI systems require sensor data for context inference. The importance of a piece of sensor data, *i.e.*, its usefulness to the current context inference process, can only be known by AmI. However, if sensor nodes can similarly know the importance of any sensor data at any given time through context exchange, the WSN can be optimized based on such knowledge. More specifically, situations such as the published sensor data on the VBs not matching with any data subscriptions from AmI while the required sensor data is delayed or lost due to network congestion can be avoided. The key idea behind this context-aware CL approach is to prioritize communications according to AmI context information. Therefore, a sensor node that anticipates its data type will become important for AmI's current context inference process can assign its next data to be published with high priority, and reconfigure its protocol parameters accordingly.

As a use-case of the proposed framework, two existing algorithms, Dynamic Reconfiguration MAC (DR-MAC) [23] protocol on the MAC layer, and Delay Aware AODV-Multipath (DAAM) [24] routing protocol on the NET layer, have been modified to incorporate the context-aware CL optimization framework proposed in Section 4. The modified protocols are referred to as context-aware DR-MAC, and context-aware DAAM, respectively.

### 5.1. Context-Aware DR-MAC

**Figure 6.**
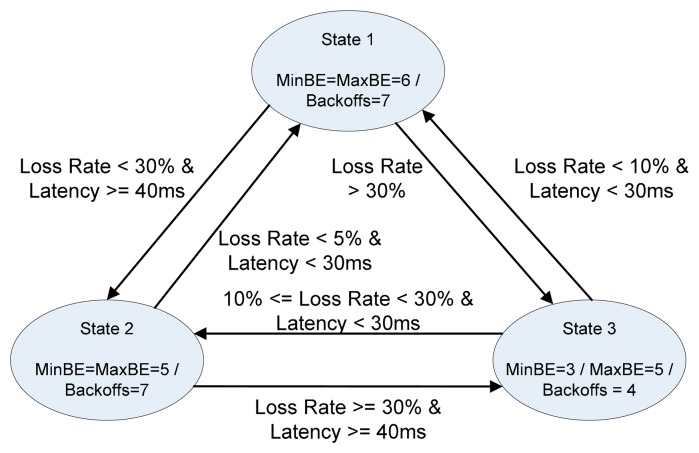
The original DR-MAC.

**Figure 7.**
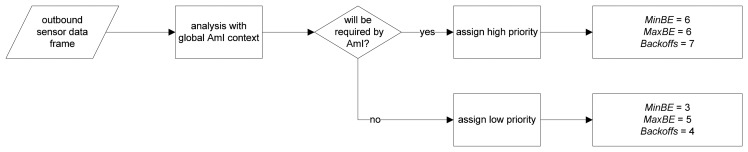
Context-aware DR-MAC.

The DR-MAC is a contention-based MAC protocol based on unslotted CSMA/CA algorithm. The original DR-MAC allows three state settings to control the number of backoffs and backoff exponential (BE) according to frame loss rate and latency, as shown in Figure 6. To incorporate AmI context information, DR-MAC is modified as shown in Figure 7.

The settings (*MinBE*, *MaxBE*, *Backoffs*) for transmitting high priority sensor data are chosen due to its having the lowest packet lost according to the results of original DR-MAC. It is important to ensure that important sensor data can arrive at its destination; otherwise it is impossible for the intelligent agents that subscribe to the sensor data to correctly perform any context inference processes. This packet loss improvement can also improve end-to-end frame latency as shown later in the evaluation section. The settings chosen for transmitting low priority sensor data is the default settings for the IEEE 802.15.4 standard.

### 5.2. Context Aware DAAM

The original DAAM is a multi-path reactive routing protocol based on the AODV routing protocol. In DAAM, multiple node-disjoint paths can be discovered by a single route discovery procedure. In addition, DAAM modifies the original AODV routing algorithm by adding the delay information for each available path. The original AODV allows the nodes, after receiving a route reply (RREP) packet, to update their routing table entities according to the following rule:
**If**
*((seq_num^d^_a_* < *seq_num^d^_b_)*
***or***
*((seq_num^d^_a_* == *seq_num^d^_b_)*
***and***
*(hop_count^d^_a_* > *hop_count^d^*
*_b_))***then** *seq_num^d^_a_:* = *seq_num^d^*
*_b_* *hop_count^d^_a_:* = *hop_count^d^*
*_b_* + *1* *next_hop*
*^d^_a_:* = *b***endif**

In AODV, when a node *a* receives a RREP packet from a one-hop neighbor node *b*, the node *a* only updates its path to the destination node *d* according to the destination sequence number (*seq_num^d^_a_* & *seq_num^d^* *_b_*) and hop counts (*hop_count^d^_a_* & *hop_count^d^* *_b_*) between node *a* and *b*. DAAM modifies AODV by keeping multiple node-disjoint paths for a pair of nodes. When routing the data packet for a particular application type, DAAM uses the following rule to allow the source node to select the best path for the packet transmission:
**If**
*(((seq_num^d^_a_* == *seq_num^d^*
*_b_)*
**and**
*(route_delay^d^_a_* > *route_delay^d^_b_)) or ((seq_num^d^_a_* < *seq_num^d^*
*_b_)*
***and***
*(route_delay^d^_a_* < *request_delay)))***then** *seq_num^d^_a_:* = *seq_num^d^*
*_b_* *hop_count^d^_a_:* = *hop_count^d^*
*_b_* + *1* *next_hop*
*^d^_a_:* = *b* *route_delay^d^_a_:* = *route_delay^d^*
*_b_***endif**

where route delay (*route_delay^d^*) represents the delay to destination node *d*, and *request_delay* represents the delay requirement of an application data packet. In this paper, DAAM has been modified to function according to the packet priority based on the user context information:
**if**
*(outbound sensor data* == *high priority)***then** *send the packet through a low delay path***else** *send the packet through a normal delay path***endif**

In this setup, a low delay path is one whose path delay is less than a threshold. Otherwise, the path is classified as a “normal” path. This delay threshold should be application-specific and defined before the nodes are deployed.

## 6. Evaluation and Analysis

### 6.1. Simulation Parameters

This section evaluates the context-aware CL design proposed in Section 5. Unless otherwise stated, the following default simulation settings are used. A WSN with 100 nodes distributed in a 10 × 10 grid topology over an area of 200 m × 200 m is simulated in OPNET. Each node has a transmission range of 20 m. A VB is formed in the centre of the network with four nodes, and 25 non-VB member nodes (*N_ami_* = 25) are randomly selected to be AmI context publishers. The remaining nodes publish their sensor data to, and subscribe to receive AmI context from, the VB periodically. The VB forwards any matched sensor data to the AmI context publishers based on their subscription messages.

The AmI context consists of *event*, *personal*, and *activity contexts* (Figure 5) whose content is randomly generated during the simulation. Six sensor data types are defined, along with five activities each requiring up to three sensor data types to be inferred. For each activity, the required number of sensor data types (e.g., 1, 2 or 3 types) and the type of sensor data (represented as Types 1–6) are randomly generated. For example, Activity one may require three sensor data types (Types 2, 3, and 6), while Activity two may require only two sensor data types (Types 1 and 5) in order to be inferred. This context structure may describe a context such as “*a blind* (personalDisability) *elderly* (personalAge) *person is walking* (activityType) *across the Queen Street* (eventLocation) *traffic junction area* (eventRadius)”.

Each AmI context publisher subscribes to sensor data types needed to generate its context, which is then published at the rate of 1 frame every five seconds with a frame size of 512 bits. The sensor data publishing settings: sensor data publishing interval (*D_freq_*) and sensor data size (*D_size_*) will be varied in this evaluation. Data rate is set to 250 kbps at 2.4 GHz. The current drawn for radio transmission, and radio reception, is set to 17.4 mA at 0 dBm, and 19.7 mA, respectively, based on MICAz's specification [25]. The AmI context publishers are mobile users who move according to a random waypoint model with a speed of 1.2 m/s and pause time of 3.6 s [26,27].

The IEEE 802.15.4 unslotted CSMA/CA and AODV are the default MAC protocol, and network routing protocol, used respectively, during the simulations. Delay threshold for context-aware DAAM is set to 1 s, which is the best setting obtained from our preliminary study. All results are the average of 10 runs over 180 s.

### 6.2. Performance Metrics

- *Delay*: the average time for a frame/packet containing sensor data published by a sensor node to arrive at the VB.
- *Throughput*: network capacity in bits per second based on published sensor data that successfully arrived at the VB.
- *Energy cost per delivered frame/packet*: ratio of total energy consumed by all nodes to the number of successfully delivered published sensor data frames to the VB.
- *Packet delivery ratio (PDR)*: ratio of the number of packets received by the VB to the total data packets sent by all sensor nodes.
- *Communication overhead*: number of control frames/packets sent during the sensor data publishing from the sensor nodes to the VB member nodes.

### 6.3. Simulation Results

#### 6.3.1. MAC Layer Results

This section evaluates the performance of three MAC protocols, DR-MAC, context-aware DR-MAC and IEEE 802.15.4 unslotted CSMA/CA, with AODV as the common network routing protocol. The results are shown for two traffic settings (normal and high traffic) as summarized in Table 2.

**Figure 8.**
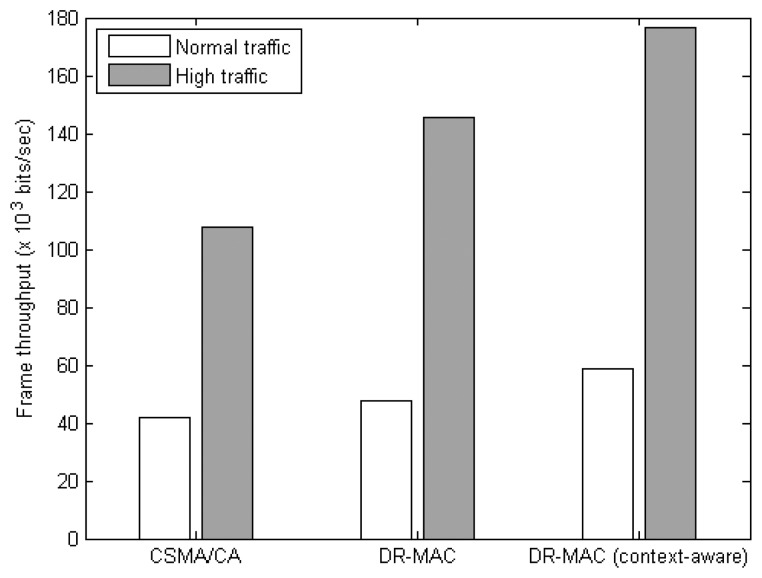
Frame throughput of MAC protocols.

Figure 8 shows the throughput results of the three MAC protocols. It is observed that the context-aware DR-MAC can achieve 22% improvements to the original DR-MAC and 36% improvements to CSMA/CA under the normal traffic. For high traffic scenario, the improvements are 22%, and 64%, respectively. The multiple backoff settings of the DR-MAC based protocols improve the frames' delivery success rate, which in turn improves their overall throughput over that of CSMA/CA. This is evidenced from the fewer number of retransmissions per frame delivered by DR-MAC-based protocols than CSMA/CA as shown in Table 3. In addition, by utilizing the AmI context information, the sensor nodes can control the frame transmissions based on their priority, and thus can further improve the overall throughput.

**Figure 9.**
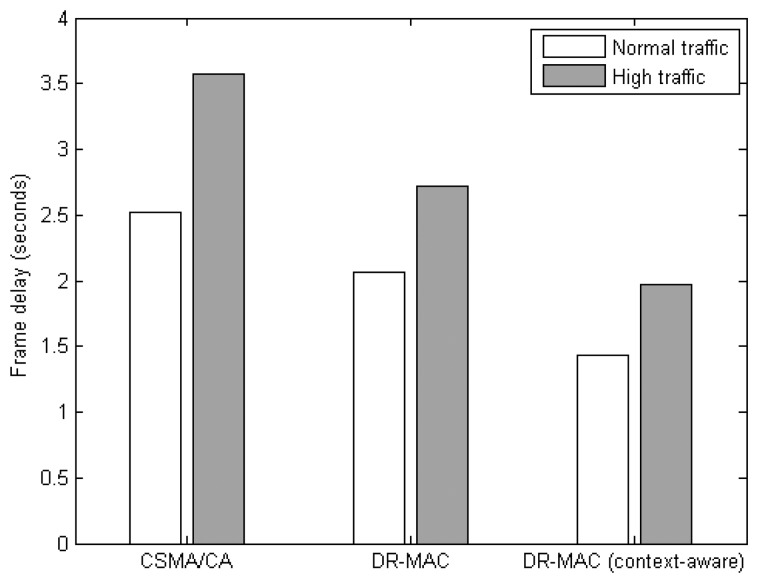
Frame delay of MAC protocols.

Figure 9 compares the frame delay among the three protocols. The context-aware DR-MAC exhibits the lowest frame delay in both normal and high traffic scenarios. The frame delay is reduced by 38%, and 30%, over CSMA/CA, and original DR-MAC, respectively, under normal traffic scenario, while it is reduced by 45%, and 28%, respectively, under high traffic scenario. The DR-MAC based protocols may increase frame delay at the MAC layer for a pair of neighbor nodes. However, it could reduce the overall end-to-end frame delay. By improving the ratio of the frames being delivered between a pair of neighbor nodes, fewer route error packets containing the link breakage messages are issued by the routing protocol of the relay nodes. Therefore, it can reduce the need of a source node to rediscover a path and retransmit the data packet to the destination node. In turn, this can reduce the overall frame delay.

**Figure 10.**
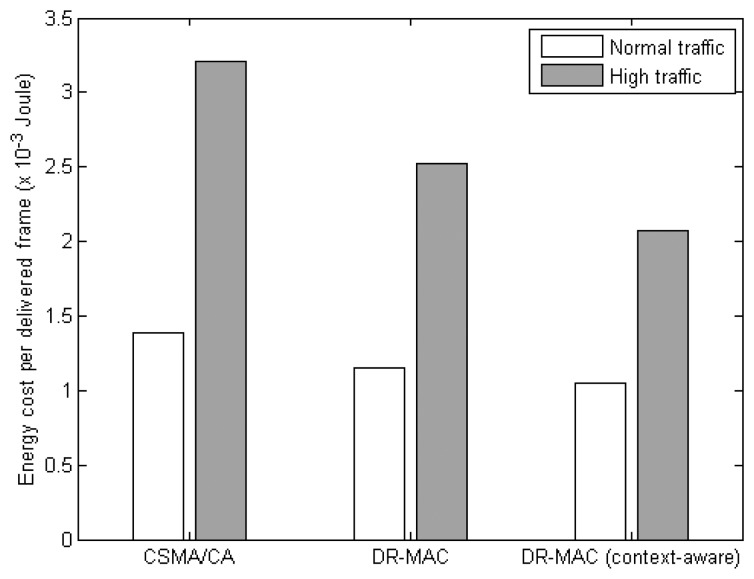
Energy cost for a successful frame delivery by MAC protocols.

Figure 10 shows the average energy cost to successfully deliver a frame to the VB. Under normal traffic scenario, the context-aware DR-MAC can achieve 25%, and 10%, improvement over CSMA/CA, and the original DR-MAC, respectively. In high traffic scenario, the improvement is 35%, and 18%, respectively. By using context information to further enhance the delivery success of the data frames, the context-aware DR-MAC incurred less energy for transmissions associated with path rediscovery and packet retransmission. Therefore, it is more energy-efficient as compared to the original DR-MAC.

#### 6.3.2. NET Layer Results

This section evaluates the performance of three network routing protocols: DAAM, context-aware DAAM and AODV, with IEEE 802.15.4 unslotted CSMA/CA as the common MAC protocol. Similarly, the results are shown for normal and high traffic settings as summarized in Table 2.

**Figure 11.**
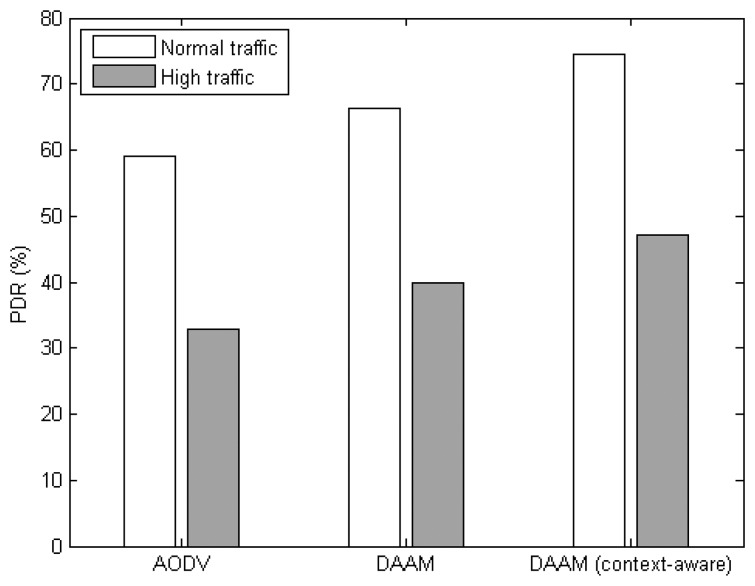
PDR of NET protocols.

Figure 11 shows the PDR of the three network routing protocols. It is observed that the context-aware DAAM can achieve 26%, and 13%, improvement to AODV and original DAAM, respectively, under normal traffic. The improvement in PDR under high traffic scenario is 44%, and 18%, respectively. In DAAM, multiple node-disjoint paths can increase the success of the packets being delivered to the VB since any of the VB member nodes can accept the incoming sensor data packets. This avoids losses due to congestion when only a single node, e.g., a cluster head, is the destination for all sensor data packets. In addition, the context-aware DAAM makes the sensor data packets traversed through paths according to their data priority; this can further improve the packets delivery to the VB.

**Figure 12.**
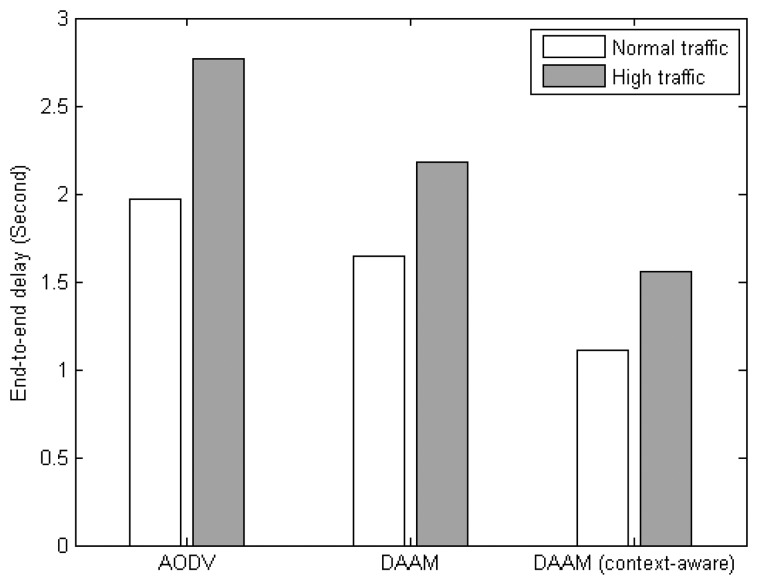
End-to-end delay of NET protocols.

Figure 12 compares the end-to-end delay among the three protocols. Under context-aware DAAM, the delay is reduced by 38%, and 32%, over AODV, and original DAAM, respectively, under normal traffic scenario, while it is reduced by 45%, and 35%, respectively, under high traffic scenario. The path diversity of DAAM significantly improves the delay performance of AODV. By further combining with AmI context information, the delay can be further reduced as low latency paths are used to transmit the high priority sensor data.

**Figure 13.**
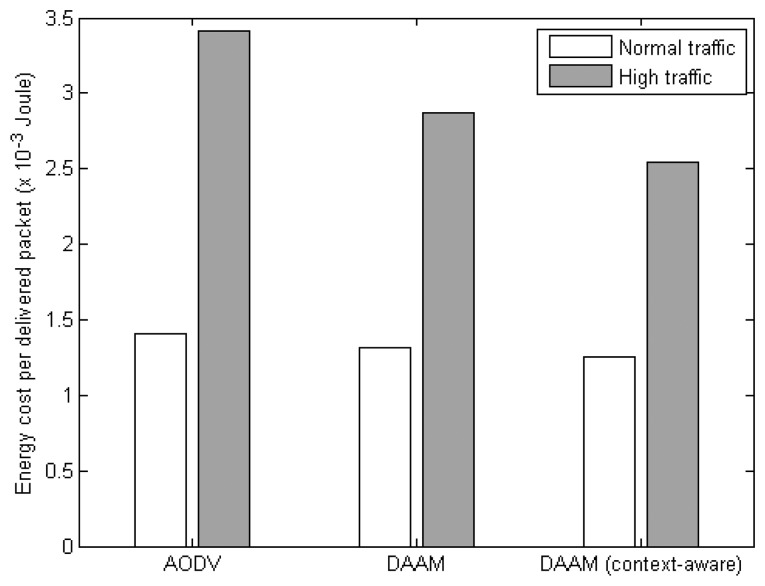
Energy cost for a successful packet delivery of NET protocols.

Figure 13 shows the corresponding energy cost. It illustrates significant energy savings under high traffic scenario where the context-aware DAAM reduces the energy cost by 24%, and 15%, over AODV, and original DAAM, respectively. Compared to AODV, which only uses a single path to transmit all the data between a pair of nodes, more available paths from the DAAM based protocols allow the data to be delivered to any member nodes of a VB. This increases the PDR, as shown in Figure 11, which in turn improves the energy efficiency.

#### 6.3.3. Results of Different Protocol Sets

This section evaluates different protocol sets based on different combinations of the MAC and NET layer protocols. A total of 9 protocol sets are defined as shown in Table 4. All sets are evaluated under normal traffic scenario with parameters as outlined in Table 2.

**Figure 14.**
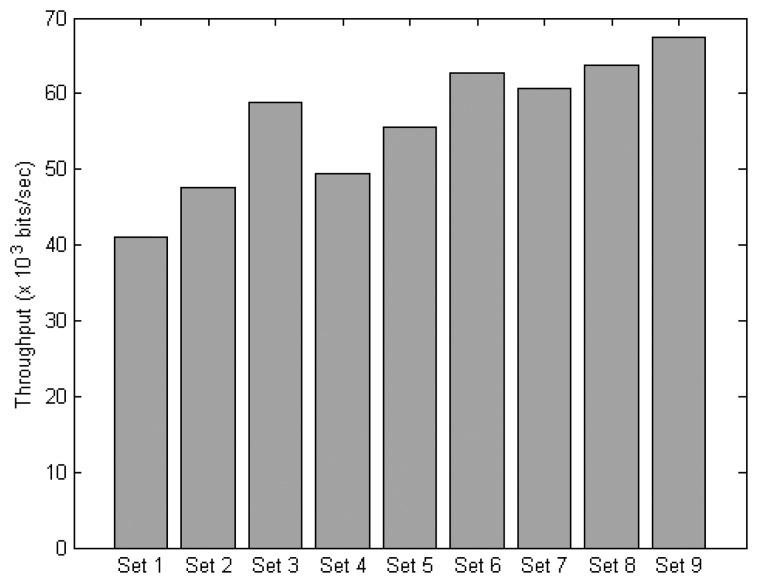
Throughput of the protocol sets.

Figure 14 shows the throughput of all the protocol sets. The largest performance differential among the protocol sets is a 64% increase in throughput by Set 9 over Set 1. Generally, it is observed that the throughput can be improved by replacing CSMA/CA with a DR-MAC based protocol. For instance, the throughput of Set 3 is improved by 36% over Set 1, 27% for Set 6 over Set 4, and 11% for Set 9 over Set 7. The less significant improvement for Set 9 over Set 7 could be due to that the context-aware DAAM has enhanced the delivery success of the sensor data packets, which leaves less room for further improvement by the context-aware DR-MAC.

**Figure 15.**
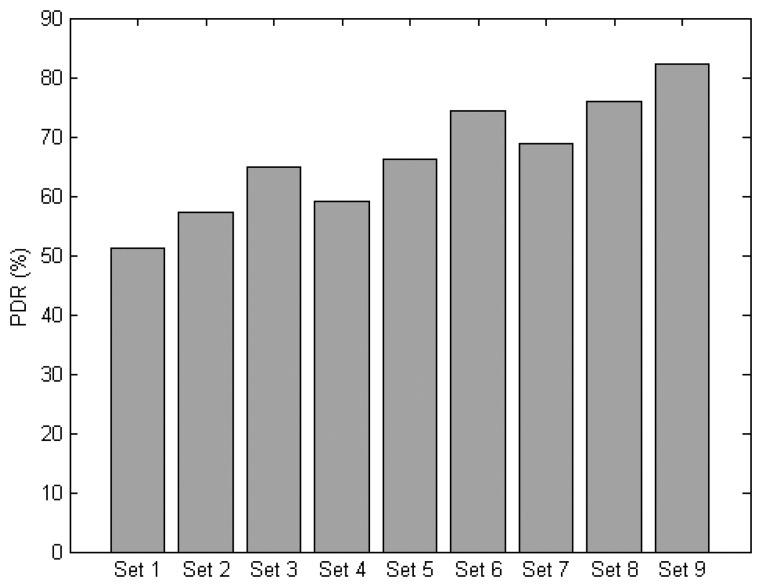
PDR of the protocol sets.

Figure 15 shows the PDR of all the protocol sets. It can be seen that the protocol sets with context-aware DAAM can achieve better PDR than the original DAAM under any MAC protocols, e.g., an improvement of 18% for Set 7 over Set 4, 14% for Set 8 over Set 5, and 11% for Set 9 over Set 6. There is a 61%, and 25%, improvement for Set 9 over Set 1, and Set 5, respectively. This result shows PDR can be improved by using AmI context information.

**Figure 16.**
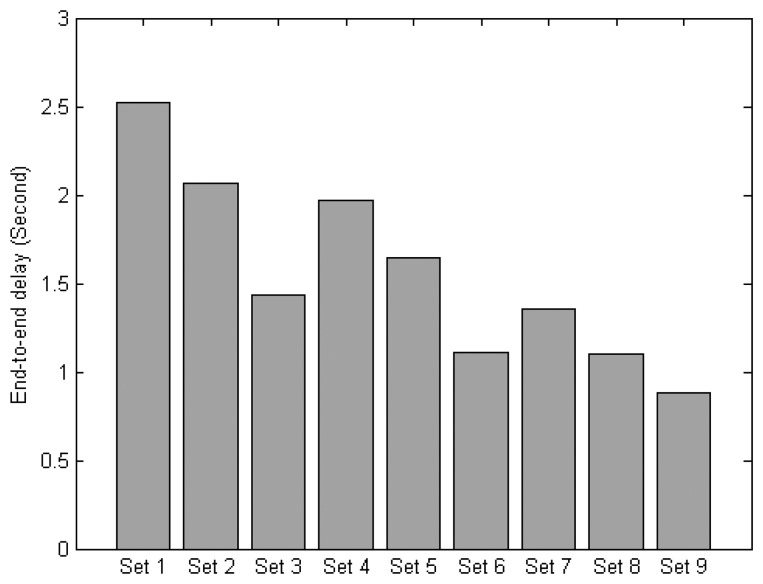
End-to-end delay of the protocol sets.

Figure 16 compares the end-to-end delay. The result shows that the delay of Set 9 is reduced by 70%, and 46%, over Set 1, and Set 5, respectively. By replacing the non-context aware MAC with a context-aware version while keeping the same routing protocol, the delay can be reduced by 30% for Set 3 over Set 2, 29% for Set 6 over Set 5, and 22% for Set 9 over Set 8. By replacing the non-context aware routing with a context-aware version while keeping the same MAC protocol, the delay can be reduced by 26% for Set 7 over Set 4, 24% for Set 8 over Set 5, and 20% for Set 9 over Set 6. These results indicate that the delay can be reduced when exploiting the context information on the MAC and network layers.

**Figure 17.**
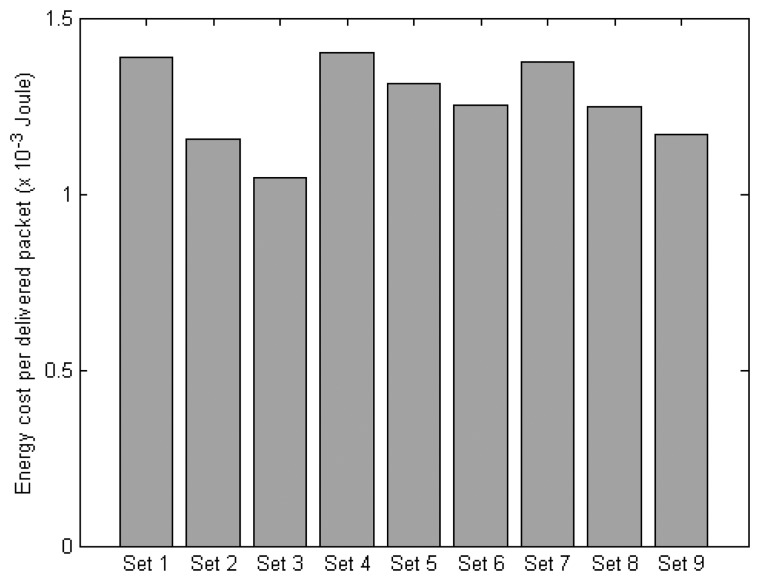
Energy cost of the protocol sets.

Figure 17 presents the average energy cost to successfully deliver a sensor data packet. In general, the protocol sets with DAAM-based routing cost more energy than those with AODV. This is due to the higher amount of energy used for transmitting control frames/packets. However, this higher energy use is partially offset by a larger number of sensor data packets delivered, resulting in only a moderate rise in the energy cost per packet delivered as compared to the protocol sets with AODV. The protocol sets with context-awareness are seen to achieve better energy efficiency. For instance, comparing Sets (3 and 2; 6 and 5; and 9 and 8) and Sets (7 and 4; 8 and 5; and 9 and 6) show that the energy cost per packet is reduced with context-awareness incorporated into the MAC, and network routing protocol, respectively.

**Figure 18.**
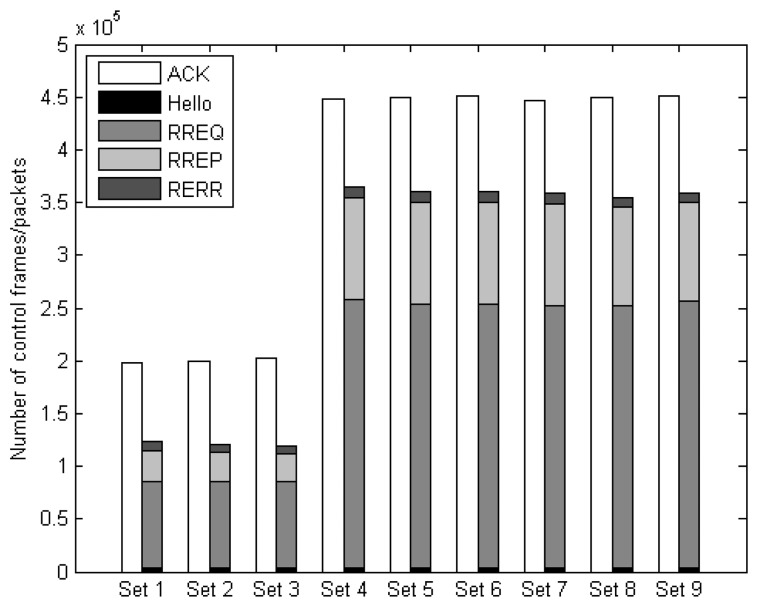
Communication overhead of the protocol sets.

Figure 18 shows the communication overhead in terms of the number of control frames (MAC layer ACK frames) and control packets (routing-related control packets). The result shows that the protocol sets with DAAM-based routing protocol have higher overhead than those with AODV. This is because unlike AODV, a route request (RREQ) packet cannot be discarded by any relay nodes during the path discovery phase in DAAM. Therefore, there are more RREQ packets being forwarded. In addition, the destination nodes have to reply to every RREQ packet received, which results in more route reply (RREP) packets as well. The DAAM-based protocol sets generate a similar amount of control packets. This is because with multiple available paths, the source node can always select another path, which can satisfy the priority requirement of the packet, to the destination node when the previous transmission fails. The route discovery procedure is performed and the associated routing overhead incurred only when none of the existing paths can be utilized for the transmission. In the simulations, the ACK frames are required for every frame transmission between a pair of nodes, where either a data or control packet from the NET layer is encapsulated into a frame. Therefore, the number of ACK frames is significantly higher than the total routing control packets.

Table 5 presents the overall rankings for all the protocol sets based on the performance metrics. The protocol set with context-aware DR-MAC and context-aware DAAM (Set 9) shows the best overall result by ranking first in throughput, PDR, and delay; second in control overhead (only two ranks in this metric); and third in energy efficiency.

#### 6.3.4. Parameter Effects on Context-Aware Protocol Set

This section evaluates the context-aware DR-MAC and context-aware DAAM protocol set (Set 9) under different traffic, node density, and node mobility settings.

##### (A) Effects of Traffic Parameters

In this section, the number of AmI user nodes (N_ami_), sensor data publishing frequency (D_freq_), and sensor data packet size (D_size_) are varied, as shown in Table 6.

**Figure 19.**
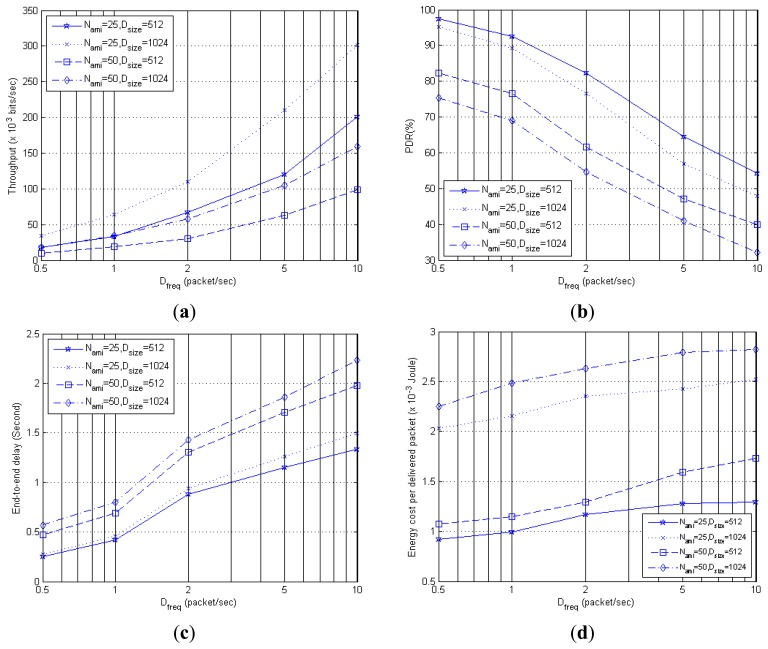
(**a**) Throughput; (**b**) PDR; (**c**) End-to-end delay; and (**d**) Energy cost performances under traffic parameter effects.

Figure 19a presents the throughput results. It shows that when D_freq_ increases, the throughput expectedly increases, as more bits are being published by sensors and delivered to VB within a given time. For some, it is interesting to note that the highest throughput with D_freq_ = 10 is even higher than the maximum data rate of 250 kbps specified in Section 6.1. This is because the throughput result is based on the total number of data bits from all data packets received by the VB divided by the simulation duration. However, our VB is not a single node, but a cooperative group of four co-located sensor nodes that share the responsibility of providing context storage and retrieval services. two or more VB nodes may receive concurrently data packets published by other non-VB sensor nodes, resulting in an overall throughput higher than that for a single node.

Similarly, when the packet size (D_size_) doubles, the throughput increases, but only by approximately 50%. This could be due to some congestion-related packet losses in the network, but the loss is not significant enough to reduce the throughput. However, when N_ami_ increases from 25 to 50 nodes while keeping the packet size constant, throughput decreases despite an increase in the amount of sensor data forwarded to the AmI nodes, *i.e.*, intelligent agents. This indicates that a serious congestion has occurred, and the network is more sensitive to an increase in the number of AmI nodes than an increase in the packet size.

Figure 19b shows the PDR results. It is observed that reasonable PDR, *i.e.*, >50% can be achieved when the sensor data publishing frequency is ≤2 packets per second. However, at higher publishing frequency, PDR decreases to below 50% for N_ami_ = 50. Similarly, doubling packet size from 512 to 1024 bits decreases the PDR. However, the PDR is not decreased proportionally by half, but up to 13%, and 20%, for N_ami_ = 25, and N_ami_ = 50, respectively. This may explain why the throughput still increases in Figure 19a when the packet size increases for a given number of AmI nodes.

Figure 19c shows the end-to-end delay results, which increase with the publishing frequency. As D_freq_ increases from 0.5 to 10 packets per second, the delay can increase by up to 5.6 times for N_ami_ = 25, D_size_ = 512, 6.1 times for N_ami_ = 25, D_size_ = 1024, 5.1 times for N_ami_ = 50, D_size_ = 512, and 3.7 times for N_ami_ = 50, D_size_ = 1024. Expectedly, the delay performances of the four settings are ordered according to the amount of sensor data bits transmitted in each setting, with the lowest delay, and highest delay, incurred by the setting N_ami_ = 25, D_size_ = 512, and N_ami_ = 50, D_size_ = 1024, respectively.

Figure 19d shows the energy cost per packet delivered increases as D_freq_ increases. This is because more energy is expended to transmit an increasing amount of sensor data, while less of these data can be delivered due to increasing network congestion. The result also shows that doubling the packet size from 512 to 1024 bits has a greater detrimental impact on the energy efficiency, *i.e.*, higher energy cost per packet, than doubling the number of AmI users from 25 to 50 nodes. This may be due to more packet reception errors and subsequently more retransmissions when long packets are used.

##### (B) Effects of Node Density and Mobility Parameters

**Figure 20.**
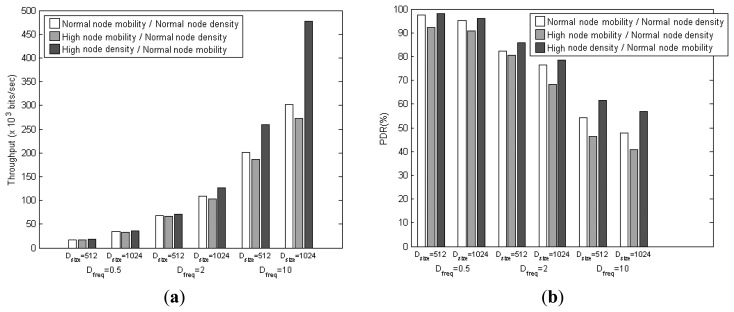

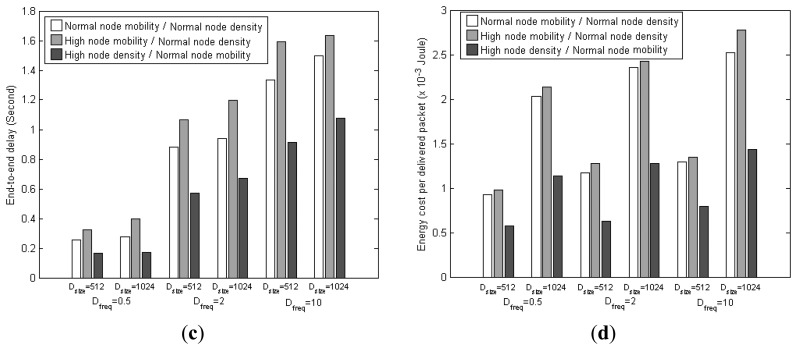
(**a**) Throughput; (**b**) PDR; (**c**) End-to-end delay; and (**d**) Energy cost performances under node density and mobility parameter effects.

In this section, the node density and node mobility are varied as shown in Table 7. Only one parameter is varied at a time (node density or node mobility, but not both). Results are obtained for N_ami_ = 25, D_freq_ = [0.5, 2, 10] packets per second, and D_size_ = [512, 1024] bits, as shown in Figure 20.

With high node mobility, the throughput and PDR expectedly decrease as more routing paths between sensor nodes/AmI users and the VB nodes become unstable. The resulting path re-discoveries inevitably increase the end-to-end delay and energy consumption. With high node density, the throughput and PDR improve due to more routing paths available. The reduction in end-to-end delay is also noticeable as routing paths are generally shorter with less number of hops, which in turn reduces the energy cost by as much as 50% compared to the case of normal node density.

#### 6.3.5. Comparison between Context-Aware and Non-Context Aware DR-MAC/DAAM Protocol Sets

This section evaluates the performance of the context-aware DR-MAC/DAAM protocol set (Set 9), and the original DR-MAC/DAAM protocol set without context awareness (Set 5). Similar to previous evaluations, results are shown for both normal and high traffic scenarios. In order to achieve further insights, additional results are shown in this section for a light traffic scenario. The traffic parameters for all three scenarios are summarized in Table 8.

**Figure 21.**
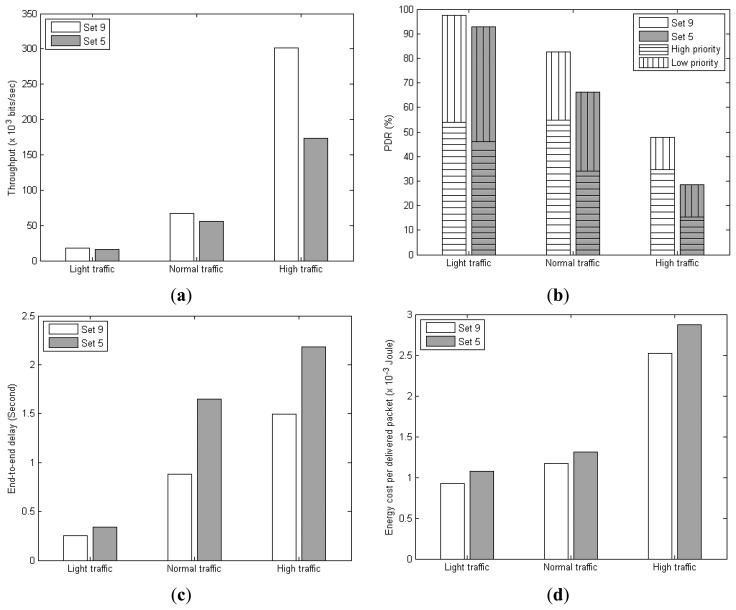
(**a**) Throughput; (**b**) PDR; (**c**) End-to-end delay; and (**d**) Energy cost comparison between context-aware and non-context aware DR-MAC/DAAM protocol sets.

Figure 21a,d present the results of the two protocol sets in terms of throughput, PDR, end-to-end delay, and energy cost per packet delivered. Clearly, context awareness can enhance the protocol performances particularly under high traffic. Protocol set 9 can achieve up to 74%, 68%, 46%, and 14% improvement over protocol set 5 in terms of throughput, PDR, end-to-end delay, and energy cost, respectively.

The improvement can be attributed to the AmI context information which is utilized: (1) for adapting the backoff behavior of the MAC protocol to enhance the success of frame delivery between neighboring node pairs; and (2) for prioritizing packets and selecting data paths with delays corresponding to the packet priority by the network routing protocol. This is evidenced from the higher proportion of high-priority data packets delivered by protocol Set 9 when AmI context information is applied, as shown in Figure 21b. This results in improved throughput, PDR, and end-to-end delay. With more sensor data packets delivered, the energy efficiency is also improved, *i.e.*, lower energy cost per packet delivered.

## 7. Conclusions

In this paper, a generic CL protocol optimization framework based on AmI context information from the application layer, in conjunction with an ontology-based context modeling and reasoning mechanism, has been proposed. This context-aware CL design provides WSN nodes with the ability to gather the AmI context for the purpose of cross-layer optimization that may involve any layer of the protocol stack. As a use case, the framework is implemented by two protocols on the MAC and NET layers for joint protocol optimizations. The backoff behavior of the MAC protocol and path selection of the network routing protocol were modified in response to AmI context information. It is shown that the resulting optimization through context awareness and cross-layer interaction can yield substantial improvements in terms of throughput, PDR, delay, and energy efficiency.

Apossible direction for future work is to extend the design of our framework to the forthcoming paradigm of the Internet of Things (IoT) where a massive number of networked devices will communicate over the Internet. Unlike WSNs, which mainly consists wireless sensor devices, IoT can encompass any devices, e.g., smart phones, smart appliances, *etc.*, interconnected through wired and/or wireless networks. As IoT is increasingly applied to realize the vision of AmI, our framework can be extended to adapt the context-aware cross-layer approach for optimizing IoT communications in AmI environments.

## Figures and Tables

**Table 1.** Context representation in different AmI scenarios.

**AmI System**	**User-Event to Detect**	**Context Expression**	**Follow-Up Action**
An ontology- and logical rule-based smart home automation system [18]	A vocal order from user to turn on a suitable light when he/she wakes up at night.	eventLocation (Bedroom) ∧ eventTime (Night) ∧ activityType(Sleeping)	Turn on bedside (not ceiling)lamp after a vocal order is received to avoid hurting the user's eyes at that moment.
	A user going to bed with main door unlocked.	eventLocation (Bedroom) ∧ activityType (Sleeping) ∧ eventObject (MainDoor, Unlocked)	Alert user to lock the main door.
An energy saving mechanism for smart office environments [19]	A user working with PC.	eventLocation (Office) ∧ activityType (Sitting) ∧ eventObject (Chair, SeatedOn)∧ eventObject (Keyboard, Manipulated)	If user-event NOT detected, switch PC and LED screen to sleep mode.
	A user at social corner under poor natural light condition (e.g. due to weather)	eventLocation (SocialCorner) ∧ eventObject (NaturalLightSensor, LowLightIntensity)	Turn on ceiling lamp and adjust its level to provide just enough light.
A context-aware and agent-based system for outdoor smart lighting [20]	A user walking or standing around a sharp corner of a street with car approaching (user is pedestrian)	eventLocation (Street) ∧ eventRadius (SharpCorner) ∧ activityType (Walking∨ Standing) ∧ eventObject (TrafficSensor, OncomingCar)	Turn on all street lamps at sharp corner to their full intensity
	Only user is at sharp corner (user is pedestrian)	eventLocation (Street) ∧ eventRadius (SharpCorner) ∧ activityType (Walking∨ Standing) ∧ eventObject (TrafficSensor, NoCar)	Turn on all street lamps at sharp corner but dim to 50% of the full intensity (enough for pedestrian's comfort)
	A user driving towards a sharp corner of a street with no pedestrians nearby (user is car driver)	eventLocation (Street) ∧ eventRadius (SharpCorner) ∧ activityType (Driving) ∧ eventObject (PedestrainSensor, NoOne)	Turn on every alternate streetlamps along the sharp corner to their full intensity
	Street is empty (a non-user event in this case, thus no personal or user activity attributes are involved)	eventLocation (Street) ∧ eventRadius (SharpCorner) ∧ eventObject (TrafficSensor, NoCar) ∧ eventObject (PedestrainSensor, NoOne)	Switch off all street lamps to save energy.
An abnormal situation monitoring and alert system for elderly care [21]	Abnormal medical situation: user is not exercising but his/her heart rate or respiration rate is very high	activityType (NotExercising) ∧ personalHealth (HeartRate ∨ RespirationRate, VeryHigh)	Alert medical consultant or caregiver about the user's abnormal situations
	Abnormal home situation: user is eating, cooking, bathing, or exercising at night while lights are off	activityType (Eating∨ Cooking ∨Bathing ∨ Exercising) ∧ eventTime (Night) ∧ eventObject (Lights, Off)	

**Table 2.** Traffic parameter settings.

**Scenario/Parameter**	**Description/Value**
Scenario 1	Normal traffic
D_freq_	2 frames per second
D_size_	512 bits
Scenario 2	High traffic
D_freq_	10 frames per second
D_size_	1024 bits

**Table 3.** Average number of retransmissions for a successful frame delivery.

	**CSMA/CA**	**DR-MAC**	**DR-MAC (Context-Aware)**
Normal traffic	1.924	1.522	1.302
High traffic	2.727	2.465	2.273

**Table 4.** Protocol sets.

**Set**	**NET Layer Protocol**	**MAC Layer Protocol**
1	AODV	CSMA/CA
2	AODV	DR-MAC
3	AODV	DR-MAC(context-aware)
4	DAAM	CSMA/CA
5	DAAM	DR-MAC
6	DAAM	DR-MAC(context-aware)
7	DAAM(context-aware)	CSMA/CA
8	DAAM(context-aware)	DR-MAC
9	DAAM(context-aware)	DR-MAC(context-aware)

**Table 5.** Performance ranking.

**Performance Metric**	**1st**	**2nd**	**3rd**	**4th**	**5th**	**6th**	**7th**	**8th**	**9th**
Throughput	Set 9	Set 8	Set 6	Set 7	Set 3	Set 5	Set 4	Set 2	Set 1
PDR	Set 9	Set 8	Set 6	Set 7	Set 5	Set 3	Set 4	Set 2	Set 1
End-to-end delay	Set 9	Set 6	Set 8	Set 7	Set 3	Set 5	Set 4	Set 2	Set 1
Control frames/packets	Set 3; Set 2; Set 1			Set 9; Set 6; Set 8; Set 7; Set 5; Set 4					
Energy efficiency	Set 3	Set 2	Set 9	Set 8	Set 6	Set 5	Set 7	Set 1	Set 4

**Table 6.** Traffic parameter settings of protocol set 9.

**Parameter**	**Value**
N_ami_	25, 50 nodes
D_freq_	0.5, 1, 2, 5, 10 packets per second
D_size_	512, 1024 bits

**Table 7.** Node density and mobility parameter settings of protocol set 9.

**Parameter**	**Description/Value**	

	**Normal**	**High**
Node density	100 nodes/ 200 × 200 m^2^	100 nodes/ 100 × 100 m^2^
Node mobility (AmI users)	1.2 m/s (walk)	4.4 m/s (run) [28]

**Table 8.** Parameter settings for context-aware and non-context aware DR-MAC/DAAM protocol sets.

**Scenario/Parameter**	**Description/Value**
Scenario 1	Light traffic
D_freq_	1 frame every 2 s
D_size_	512 bits
Scenario 3	High traffic
D_freq_	2 frames per second
D_size_	512 bits
Scenario 3	High traffic
D_freq_	10 frames per second
D_size_	1024 bits
